# Effect of Calcium-enriched Mixture (CEM) cement on increasing mineralization in stem cells from the dental pulps of human exfoliated deciduous teeth

**DOI:** 10.15171/jpid.2018.036

**Published:** 2018-12-19

**Authors:** Rezvan Rafatjou, Iraj Amiri, Atousa Janeshin

**Affiliations:** ^1^Department of Pediatric Dentistry, School of Dentistry, Hamadan University of Medical Sciences, Hamadan, Iran; ^2^Department of Anatomy, Hamadan University of Medical Sciences, Hamadan, Iran; ^3^Dental Sciences Research Center, Department of Pediatric Dentistry, School of Dentistry, Guilan University of Medical Sciences, Rasht, Iran

**Keywords:** Calcium-enriched mixture (CEM) cement, deciduous tooth, dental pulp, stem cells

## Abstract

***Background.*** Stem cells isolated from human exfoliated deciduous teeth (SHED) are highly capable of proliferation and differentiation into odontogenic, osteogenic, adipose tissue and neural cells. The aim of this study was to investigate the effect of CEM cement on increasing mineralization in stem cells of exfoliated deciduous teeth.

***Methods.*** Dental pulps were isolated from extracted exfoliating primary teeth and immersed in a digestive solution. The dental pulp cells were immersed in α-MEM (modified culture medium) and 10% fetal bovine serum (FBS) was added. The culture cells were used for mineral deposit formation after the third passage. The cells were cultured in osteogenic cell culture medium in the control group and in osteogenic culture medium supplemented with CEM cement in the case group. Alizarin red staining was used to evaluate the mineral deposit formation on day 21. Statistical significance was determined with t-test.

***Results.*** Quantification of alizarin red staining showed that cells exposed to CEM cement induced more mineralized nodules (P=0.03).

***Conclusion.*** Mineral deposit formation in SHEDs was stimulated by CEM cement. Based on these data it might be suggested that CEM could improve osteoblastic differentiation.

## Introduction

During tooth development, interactions between epithelial and mesenchymal cells lead to differentiation of odonotoblasts which deposit specialized mineralized dentin.^1^ Cell division and secretion of odontoblasts in the adult pulp are limited, but after dentinal damage these processes might be re-activated, resulting in the formation of tertiary dentin, including reactive and reparative dentin. In response to minor injuries, primary odontoblasts remain intact and reactionary tertiary dentinogenesis occurs but reparative dentin is formed after severe injuries that lead to odontoblast death.^2^ These data demonstrate that postnatal dental pulp contains cells which can have stem cell properties.^3^

Mesenchymal stem cells (MSCs) isolated from various adult tissues are common candidates for tissue engineering. These cells are highly proliferative and multipotent with the capability to differentiate into several cell types; therefore, they have been considered as a potential cell source for bone regenerative therapies.^4,5^ Dental pulp stem cells (DPSCs) are considered a good source of multipotent mesenchymal stem cells and have the ability to differentiate into odontoblasts, osteoblasts, chondrocytes, myocytes and adipocytes.^6^ Dental stem cells can be isolated from several dental sources, including DPSCs,^3^ stem cells from human exfoliated deciduous teeth (SHEDs),^7^ periodontal ligament stem cells (PDLSCs),^8^ stem cells from the apical papilla (SCAPs)^9^ and dental follicle precursor cells (DFSCs)^10^.

SHEDs have the capacity for self-renewal and multilineage differentiation and can differentiate into osteogenic and odontogenic cells, adipocytes and neural cells.^11^ Previous studies showed that the proliferation rate of SHEDs was greater than DPSCs and bone marrow-derived mesenchymal stem cells (BMMSCs).^12^

SHEDs have several specific advantages over dental pulp stem cells. One of advantages is that they can be easily isolated from a readily accessible tissue source. Lack of tissue destruction at donor site, reduction or elimination of discomfort and pain and the possibility of obtaining pulp cells from younger patients are other advantages of these cells.^13^ Therefore these cells can be regarded as valuable sources of stem cells for tissue engineering and cell replacement therapy in different fields such as bone regeneration.^7^ Another field of research following the isolation of stem cells from the tissue source is to find appropriate materials to add to the cell culture medium so that the most efficient material for the proliferation and differentiation of stem cells is introduced.

Calcium-enriched mixture (CEM) cement, a new material which has been introduced to dentistry, comprises water-soluble calcium and phosphate (calcium hydroxide,calcium oxide, calcium sulfate, calcium phosphate,calcium carbonate and calcium silicate), and forms hydroxyapatite after setting.^14^ CEM can also stimulate hard tissue healing.^15^ Several investigations with different cell culture systems have demonstrated that CEM has low cytotoxicity and high biocompatibility.Fresh CEM is water-soluble and has also demonstrated good cell viability values.^15^ This study was designed to investigate the effect of CEM cement on mineral deposit formation (alizarin red staining) of stem cells of exfoliated deciduous teeth.

## Methods

Exfoliated deciduous teeth were collected from 10 systematically healthy patients (6‒11 years old); the teeth were free of carious lesions. Prior to extraction, the patients received oral health education, professional prophylaxis was carried out for each patient, and they used 0.2% chlorhexidine mouthwash for one minute. Immediately after extraction, the teeth were immersed in basic medium (Dulbecco’s Modified Eagle’s Medium, DMEM), and transported to the laboratory. Under aseptic conditions, the dental pulp was extracted with a dental broach, and was immersed in a digestive solution containing 100 U/mLof penicillin, 100 mg/mLof streptomycin, 500 mg/mLof clarithromycin and 0.25 mg/mL ofamphotericin B in 4 mL of 0.1 M phosphate-buffered saline (PBS) with the addition of 4 mg/mL ofdispase and 3 mg/mL of type I collagenase for 1 hour at 37°C. The solution was filtered through 70μm Falcon strainers (Falcon; Fisher, USA). After filtration, the cells were immersed in α-MEM (minimum essential medium) (Gibco; Germany), supplemented with 10% fetal bovine serum (FBS) (Gibco; Germany), 100μm 2P-ascorbic acid, 2 mM L-glutamine, 100 μg/mLof streptomycin and 100 U/mLof penicillin. The cell suspension was centrifuged and placed in 25-mL flasks. The flasks were maintained in a 5% CO_2_ atmosphere at 37°C, and the culture medium was refreshed twice a week.^16^

### Mineralization assay

After the third passage, the cells were isolated from the culture plate by using 25% trypsin-EDTA and cultured for 24 hours. When the cell density reached 80%, they were used for osteogenic differentiation.

As the control group, the cells were cultured in osteogenic cell culture medium containing α-MEM, 10% FBS, 50 μg/mL of L-ascorbic acid 2-phosphate, 50 mM β-glycerophosphate, and 0.1 μM dexamethasone (Sigma). As the case group, the cells were cultured in osteogenic culture medium supplemented with fresh CEM cement.The differentiation process was repeated 12 times. The medium was replaced with fresh medium every 3‒4 days. After 21 days of culture, the mineralization was analyzed by alizarin red staining (ECM 815; Millipore, USA). For alizarin red staining, the plates were rinsed three times with FBS and fixed with 10% formaldehyde. Then, the cultures were stained with alizarin red solution for 20 minutes at room temperature. Excess dye was removed in case of over-staining by washing three times with distilled water. Digital images were captured using an inverted microscope. The total mineralized tissues were counted according to the manufacturer's instructions of alizarin red. Forquantitative evaluation of alizarin red staining, the OD405 (optical density) values of a set of known alizarin red concentrations were determined and compared with the obtained values.^17,18^

All the statistical calculations were performed with SPSS18.0, using t-test. A 𝑃-value ≤ 0.05 was considered significant. For the analysis of normal distribution of data, non-parametric Kolmogorov-Smirnov test was used.

## Results

### Morphologic findings

**Figure 1:**
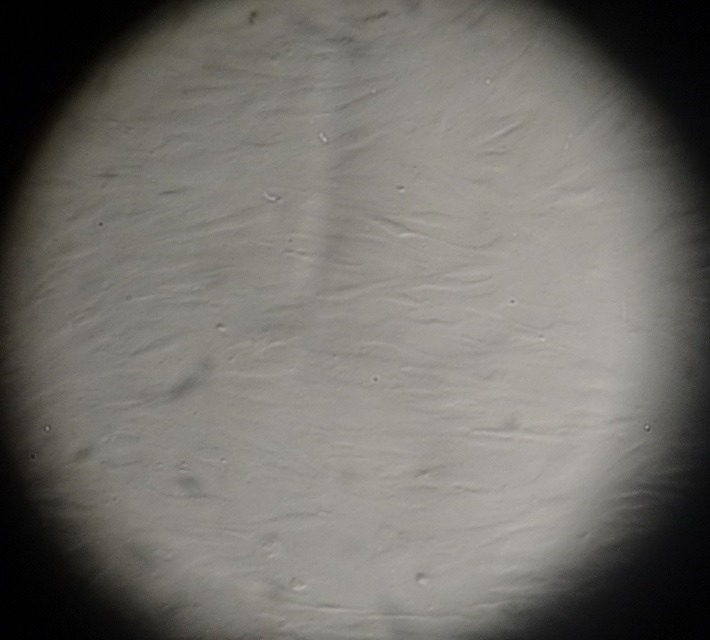
Dental pulp stem cells of deciduous teeth on day 3 (×100 magnification).

The medium was observed under an inverted light microscope on the first, third and seventh day and in the third passage. SHEDs exhibited fibroblast-like morphology and during cell passage the morphology of the cells remained constant (Figure 1).

### Alizarin red staining

**Figure 2:**
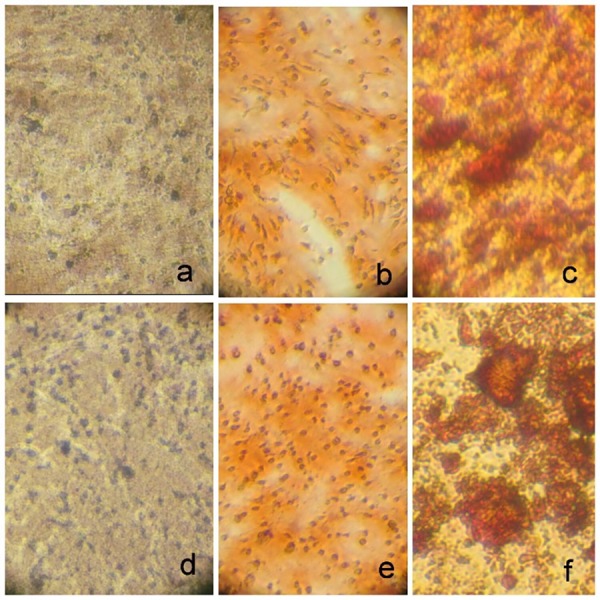
Alizarin red staining of mineralized deposits without (A, B, C) and with (D, E, F) CEM cement. Osteogenic differentiation before staining at ×40 (A and D), after staining at ×40 (B and E) and after staining at ×400 magnifications (C and F).

The effect of CEM cement on increasing mineralization in SHEDs was analyzed by alizarin red staining. Quantification of alizarin red staining showed that cells exposed to CEM cement induced more mineralized nodules (P=0.03)(Table 1, Figure 2).

**Table 1:** The t-test results of alizarin red staining

**Group**	**Number of** **examination**	**Mean** **(**μ**M/Ml)** ± **SEM**	**P-value**
**Case**	8	85.07‏±358.33	P=0.03
**Control**	8	73.32‏±673.21	

## Discussion

In this study, cells were isolated from the normal dental pulp tissue of exfoliated deciduous teeth of healthy patients. The osteogenic differentiation of stem cells is a complex process, which is influenced by multiple materials.^19^ CEM cement has been recently introduced as a useful dental material for various clinical indications. The ability of CEM cement to release calcium ions during setting has been associated with its biocompatibility and the subsequent binding of calcium with phosphorus to form hydroxyapatite crystals. This process facilitates hard tissue healing.^20^ This study aimed to isolate dental pulp stem cells from exfoliated deciduous teeth and evaluate the effects of CEM cement on mineralization of these cells.

SHEDs are postnatal stem cells that have extensive proliferation and differentiation potentials. Mesenchymal stem cellshave been extensively characterized in vitroby the expression of markers such as STRO-1, CD146 or CD44.^18^ One limitation of this study was that we could not carry out flow cytometric analysis of cell surface due to financial and technical resources limitations.

Deciduous teeth therefore may be a suitable resource of stem cells to induce bone regeneration.^7^ Regeneration of damaged bone is an important topic; bone tissue engineering methods consist of three elements: a donor cell source, osteoinductive growth factors and a three-dimensional scaffold.^21^ Therefore, a suitable osteogenic factor for bone tissue engineering is critical for the success of osteogenic differentiation. Therefore, many studies have been undertaken to find appropriate methods for achieving this aim.^22^

Based on previous studies, differentiation of mesenchymal stem cells such as DPSC and SHED into osteoblast-like cells is induced by dexamethasone, ascorbic acid, B-glycerophosphate and 1α, 25 (OH) 2D3.^19,23^ Osteoblastic differentiation in DPSCs and DFPCs was stimulated by both 1α,25(OH)2D3 and 25OHD3.^24^ Osteoblast differentiation in SHEDs was also stimulated by 1α,25(OH)2D3.^19^

In the present study,to evaluate the effect of CEM cement on mineralization after the third passage, the cells were cultured in routine osteogenic culture medium with and without the addition of CEM cement. After three weeks, alizarin red staining was measured.

Alizarin red staining is a marker of osteogenic differentiation. Khanna et al reported that dental follicle stem cells and dental pulp stem cells formed more mineralized nodules when treated with vitamin D3 metabolites in osteogenic differentiation culture.^24^ Mojarad et al showed that SHEDs formed more mineralized matrix in cell culture treated with 1,25(OH)2D3.^19^ Here we showed that CEM increased alizarin red staining in cell culture. Therefore, the response of deciduous dental pulp stem cells was the same when treating with CEM and vitamin D metabolites.

TEGDMA (0.25mM, 0.1mM) and HEMA (0.5mM and 0.1mM) reduce the alizarin red staining, resulting in osteogenic culture differentiation,^17^ which is different from the effect of CEM and vitamin D_3_.

## Conclusion

This study demonstrated that mineral deposit formation in SHEDs was stimulated by CEM cement. Based on these data it might be suggested that CEM could improve osteoblastic differentiation. Further research is needed to suggest CEM as a useful therapeutic agent when osteogenic differentiation is desired.

## Acknowlegements

The authors would like to thank Dr Motahareh Mortazavi for her helpful suggestions in this study.

## Authors’ contributions

AJ and RR conceived the idea. AJ and IA carried out the experiments, and collected and analyzed the data. AJ and RR prepared the manuscript. All authors have read and approved the final manuscript.

## Funding

This study was financially supported by Hamadan University of MedicalSciences.

## Competing interests

The authors decalre that they have no competing interestswith regards to the authorship and/or publication of this article.

## Ethics approval

The study protocol was approved by the Research Ethics Committee of Hamadan University of Medical Sciences.
